# Nephrotic Syndrome and Idiopathic Membranous Nephropathy Associated with Autosomal-Dominant Polycystic Kidney Disease

**DOI:** 10.1100/tsw.2011.94

**Published:** 2011-05-05

**Authors:** Ramón Peces, Jorge Martínez-Ara, Carlos Peces, Mariluz Picazo, Emilio Cuesta-López, Cristina Vega, Sebastián Azorín, Rafael Selgas

**Affiliations:** ^1^ Servicio de Nefrología, Hospital Universitario La Paz, IdiPaz, Madrid, Spain; ^2^ Servicio de Anatomía Patológica, Hospital Universitario La Paz, Madrid, Spain; ^3^ Servicio de Radiología, Hospital Universitario La Paz, Madrid, Spain; ^4^ Area de Tecnologías de la Información, SESCAM, Toledo, Spain

**Keywords:** ADPKD, glomerulonephritis, membranous nephropathy, mycophenolate mofetil, nephrotic syndrome, proteinuria

## Abstract

We report the case of a 38-year-old male with autosomal-dominant polycystic kidney disease (ADPKD) and concomitant nephrotic syndrome secondary to membranous nephropathy (MN). A 3-month course of prednisone 60 mg daily and losartan 100 mg daily resulted in resistance. Treatment with chlorambucil 0.2 mg/kg daily, low-dose prednisone, plus an angiotensin-converting enzyme inhibitor (ACEI) and an angiotensin II receptor blocker (ARB) for 6 weeks resulted in partial remission of his nephrotic syndrome for a duration of 10 months. After relapse of the nephrotic syndrome, a 13-month course of mycophenolate mofetil (MFM) 2 g daily and low-dose prednisone produced complete remission for 44 months. After a new relapse, a second 24-month course of MFM and low-dose prednisone produced partial to complete remission of proteinuria with preservation of renal function. Thirty-six months after MFM withdrawal, complete remission of nephrotic-range proteinuria was maintained and renal function was preserved. This case supports the idea that renal biopsy is needed for ADPKD patients with nephrotic-range proteinuria in order to exclude coexisting glomerular disease and for appropriate treatment/prevention of renal function deterioration. To the best of our knowledge, this is the first reported case of nephrotic syndrome due to MN in a patient with ADPKD treated with MFM, with remission of proteinuria and preservation of renal function after more than 10 years. Findings in this patient also suggest that MFM might reduce cystic cell proliferation and fibrosis, preventing progressive renal scarring with preservation of renal function.

## INTRODUCTION

Autosomal-dominant polycystic kidney disease (ADPKD) is an inherited disorder characterized by the development and growth of cysts in the kidneys. Mild proteinuria, <1 g/day, is a common finding in patients with ADPKD[1]. However, association with nephrotic syndrome is considered to be rare in this disorder[2,3,4]. Therefore, massive proteinuria in ADPKD patients should raise the suspicion of a complicating glomerular disease. We report a patient with ADPKD who developed nephrotic syndrome and renal biopsy showed membranous nephropathy (MN). The patient was treated with mycophenolate mofetil (MFM) and 36 months after MFM withdrawal, complete remission of nephrotic-range proteinuria was maintained and renal function was well preserved.

## CASE REPORT

A 38-year-old man with ADPKD presented in April 2000 with a 4-week history of generalized edema. He had hyperlipidemia, hypoalbuminemia, and proteinuria of 11.8 g/24 h. Serum creatinine was 1 mg/dl (76.26 µmol/l) and creatinine clearance was 109 ml/min/1.73 $m^2$. Tests for HBsAg, HCV-ab, HIV, VDRL, and ANA were negative. Serum C3 and C4 levels were normal. Abdominal ultrasound (US) and computed tomography (CT) showed both kidneys with multiple small cysts, with the right kidney measuring 11.7 cm in length with a 5-cm cyst at the superior pole, and the left kidney measuring 13 cm in length with a 1.5-cm sinusal cyst. At this point, a US-guided renal biopsy was performed. Light microscopy revealed 12 glomeruli showing normal size and cellularity, global diffuse capillary wall thickening with subepithelial fuchsinophilic deposits, and widespread epimembranous spikes with methenamine silver staining (Fig. 1A). Immunofluorescence (IF) studies showed global diffuse fine granular deposition of IgG (3+), C3 (2+), and fibrinogen (1+) along the peripheral capillary loops (Fig. 1B). Based upon these findings, stage II idiopathic MN was diagnosed, and treatment started with oral prednisone (60 mg/day) and losartan (100 mg/day). After 3 months of steroid treatment without response, prednisone was tapered. In January 2001, treatment with chlorambucil 0.2 mg/kg/day and prednisone 20 mg/day for 6 weeks, together with an angiotensin-converting enzyme inhibitor (ACEI) (enalapril) and an angiotensin II receptor blocker (ARB) (telmisartan), resulted in partial remission of the nephrotic syndrome. Ten months after chlorambucil withdrawal, relapse of the nephrotic syndrome occurred. In December 2001, he received treatment with MFM (2 g/day) and prednisone (20 mg/day), which were maintained for 13 months. The proteinuria gradually decreased to <0.5 g/24 h and renal function remained stable. Forty-four months after MFM withdrawal, proteinuria relapsed. Then, in December 2005, MFM (2 g/day) and prednisone (20 mg/day subsequently tapered) were reinitiated. Hypercholesterolemia was diagnosed and treated with atorvastatin 20 mg daily. The proteinuria gradually decreased to about 1 g/24 h and MFM was maintained for 24 months. In May 2008, abdominal magnetic resonance imaging (MRI) showed conserved kidney form with multiple small cysts (4–10 mm), the right kidney with a 6-cm cyst at the superior pole and the left kidney with several 2- to 3.5-cm sinusal cysts. The liver also displayed multiple cysts. In February 2009, proteinuria ranged from 0.7 to 1.4 g/24 h, serum creatinine was 1.1 mg/dl (83.89 µmol/l), and creatinine clearance was 93 ml/min/1.73 $m^2$. In October 2010, 36 months after MFM withdrawal, proteinuria was 0.4 g/24 h, serum creatinine was 1.1 mg/dl (83.89 µmol/l), and creatinine clearance was 114 ml/min/1.73 $m^2$. Serum total cholesterol levels remained controlled with treatment, with atorvastatin 20 mg daily. His treatment with both an ACEI and an ARB is continuing. The clinical evolution is summarized in Table 1.

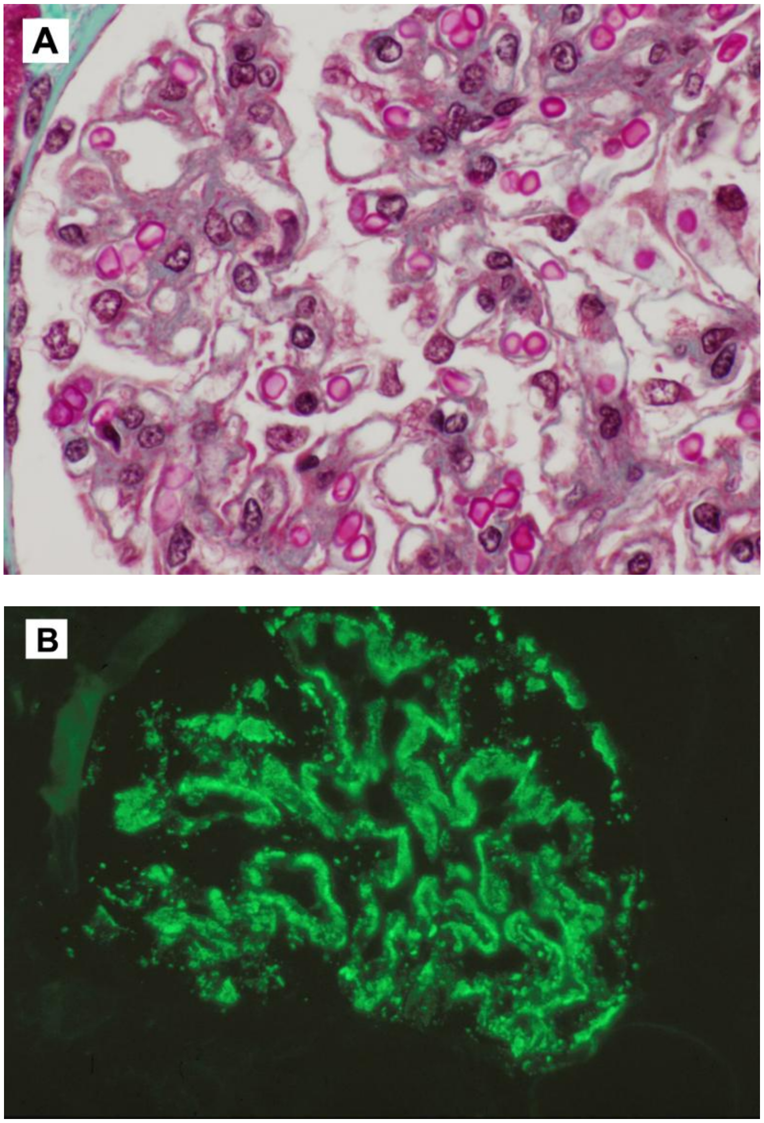

**FIGURE 1**. (A) Light microscopy image showing segmental subepithelial fuchsinophilic deposits (hematoxylin eosin). (B) IF showing diffuse granular deposits of IgG.

## DISCUSSION

Although mild proteinuria has been reported to occur in ADPKD[1], nephrotic syndrome is exceptional and its presence should raise the possibility of complicating glomerular disease[2,3]. Hiura et al.[3] reported an ADPKD patient with IgA nephropathy and reviewed 21 cases of ADPKD, including cases in the English and Japanese literature, in which the renal lesion was evaluated by histopathologic studies. Of the 21 patients, focal segmental glomerulosclerosis and minimal change nephropathy were the dominant diagnoses, with five patients each. Next in frequency was MN, with three patients, and IgA nephropathy, with two patients. Six other patients had a variety of diagnoses (diabetes, amyloidosis, mesangioproliferative and crescentic glomerulonephritis). There is only one report of ADPKD with nephrotic syndrome secondary to lupus nephritis[4]. Sar et al.[5] reported a new case of secondary amyloidosis and ADPKD. D'Cruz et al.[6] reported a case of ADPKD with nephrotic-range proteinuria and

**TABLE 1**
**Evolution of an ADPKD Patient with Nephrotic Syndrome and MN**

| Date | Proteinuria g/24 h | Cr mg/dl (µmol/l) | Cr Cl ml/min/1.73 $m^2$ | Treatment and Outcome |
|---|---|---|---|---|
| June 2000 | 11.8 | 1.0 (76.26) | 109 | Prednisone (3 months)<br>Resistance |
| January 2001 | 11.5 | 1.2 (91.51) | 105 | Chlorambucil (6 weeks) + Prednisone<br>Partial remission |
| December 2001 | 26.9 | 1.2 (91.51) | 104 | Mycophenolate (13 months) + Prednisone<br>Complete remission |
| December 2005 | 6.2 | 1.0 (76.26) | 106 | Mycophenolate + Prednisone<br>Partial remission |
| October 2006 | 2.2 | 0.9 (68.63) | 136 | Mycophenolate<br>Partial remission |
| October 2007 | 1.0 | 1.1 (83.89) | 109 | Mycophenolate<br>Partial-complete remission |
| February 2009 | 1.4 | 1.1 (83.89) | 93 | Enalapril + Telmisartan<br>Partial-complete remission |
| October 2009 | 1.7 | 1.2 (91.51) | 95 | Enalapril + Telmisartan<br>Partial-complete remission |
| January 2010 | 0.3 | 1.3 (99.14) | 104 | Enalapril + Telmisartan<br>Complete remission |
| April 2010 | 0.7 | 1.3 (99.14) | 112 | Enalapril + Telmisartan<br>Complete remission |
| October 2010 | 0.4 | 1.1 (83.89) | 114 | Enalapril + Telmisartan<br>Complete remission |

histologically proven poststreptococcal diffuse proliferative glomerulonephritis. Recently, a case of steroid-sensitive nephrotic syndrome[7] and a new case of MN have been reported[8] in children. Since there is no apparent pathogenic link between ADPKD and the different causes of nephrotic syndrome that have been reported in ADPKD patients, it suggests that they are likely coincidental diseases. The evaluation of the data of these patients suggests that the presence of concomitant glomerular disease may accelerate the course of ADPKD toward end-stage renal disease (ESRD) and may require specific treatment to control the proteinuria. Importantly, proteinuria and hypertension are independent risk factors for the development of ESRD in ADPKD[1]. At present, it is impossible to assess the potential benefit of measures leading to a decrease in the proteinuria in the progression of ADPKD toward ESRD. In addition, currently there is no effective treatment available to retard cyst growth and to prevent progression to ESRD in patients with ADPKD. Thus, although spontaneous remission of the glomerulopathy cannot be completely excluded in our ADPKD patient, the nephrotic syndrome was apparently responsive to treatment with MFM, so the patient could be considered a lucky responder to MFM. Furthermore, he maintained complete remission of nephrotic-range proteinuria and stable renal function after more than 10 years. One possible explanation for this finding is that MFM might reduce cystic cell proliferation, angiogenesis, and fibrosis, preventing progressive renal scarring[9,10] with preservation of renal function. Although the explanation about possible beneficial influences of MFM on the clinical evolution of ADPKD in this patient can appear speculative, MFM has proven highly effective in reducing renal cystogenesis and loss of renal function in an animal model of polycystic kidney disease[10]. However, the effect of MFM in kidney volume and renal function of ADPKD patients has not been established.

The evaluation of the data of these patients also revealed that only anecdotal case reports of ADPKD received a US- or CT-guided percutaneous renal biopsy[4,8], while the remaining patients received an open surgical biopsy[2,3,6]. The renal biopsy is an invaluable tool in the diagnosis, prognosis, and management of patients with kidney disease. The cost-benefit ratio reaches high levels because it is able to define abnormal kidney situations to a profound extent. Uncertain nephropathies, proteinuria and glomerular-origin hematuria, other urine sediment abnormalities, interstitial disease, and transplanted patients are among the situations recognized as definitely benefiting from kidney biopsy. Occasionally apparent simple nephropathies are present as complex situations associating renal syndromes pathogenically diverse. This is the case of ADPKD associated with the nephrotic syndrome. It opened the necessity of performing a kidney biopsy in a cystic nephropathy. In this case, just the complementary histological study was able to define the glomerular situation in appearance pathogenically separated from the principal process, the cystic degenerative change of renal parenchyma. The percutaneous renal biopsy has been established as a safe and effective method of obtaining renal parenchyma. With newer technology, the use of real-time US guidance and automated biopsy needles has improved the rate of successful diagnosis in over 95% of cases[11]. US can localize the desired lower pole site, determine renal size, and detect the presence of large cysts that might necessitate using the contralateral kidney. CT-guided renal biopsy is an alternative when the kidneys cannot be well visualized, as with marked obesity or small echogenic kidneys. Complications, although rare, may occur and the majority of these are related to bleeding. However, there are absolute and relative contraindications, such as solitary kidney, uncontrolled arterial hypertension, hemostasis disorders, renal artery aneurysm, Jehovah witness, percutaneous needle renal biopsy failure, morbid obesity, and noncollaborative patients. In patients with contraindications to the percutaneous approach, alternative methods of obtaining tissue have been attempted. In these situations, open renal biopsy is the option through flank or posterior incision. Because multiple bilateral cysts is listed as a relative contraindication to percutaneous renal biopsy, due to the presumed risk of complications and difficulties in obtaining suitable tissue for diagnosis, a majority of polycystic kidney disease patients received an open surgical biopsy[2,3,4]. This literature consisted mostly of case reports and there are no published trials. Only in some few cases of polycystic kidney disease, including our patient, US- or CT-guided renal biopsy was performed without complications[6]. However, the experience with this practice is too limited to recommend it safely for widespread practice. Although the open, or surgical, renal biopsy has been performed for more than 40 years as the standard procedure in patients with contraindications to the percutaneous approach, alternative methods that may be less invasive have been pursued. These include laparoscopic, transurethral, or transvenous renal biopsy[11,12,13,14,15]. The transvenous biopsy technique uses the native vessels as an alternate route to an organ. The tissue specimen is obtained from inside out by passing the needle through the vein wall into the surrounding parenchyma[12,13,14,15,16]. The theoretical advantages are that bleeding occurs back into the vein; the needle is directed away from, rather than toward, the larger organ vessels; and there is less likelihood of capsular perforation. Disadvantages include smaller sample size (and thus decreased diagnostic yield) and limited availability of technical expertise. This procedure may be an option in selected circumstances when the appropriate expertise is available. There were no published trials with this procedure in patients with polycystic kidney disease. Laparoscopic renal biopsy can be performed via a laparoscopic retroperitoneal (retroperitoneoscopy)[17,18,19] or transperitoneal approach[20]. These methods of biopsy are advantageous in that the kidney is positively identified, and that cortical biopsy and hemostasis are achieved under direct vision. In addition, because laparoscopic retroperitoneal renal biopsy is minimally invasive, recovery and convalescence are short for most patients[17,19], and the technique can be performed on an outpatient basis. The retroperitoneal endoscopic approach has gained popularity both worldwide and in our country, including indications in pediatric cases[21,22]. In fact, the retroperitoneoscopy or transperitoneal approach renal biopsy is now the preferred procedure for pediatric patients[23]. A new approach combining laparoscopy and percutaneous needle biopsy has been proposed[24]. This approach combines the advantages of percutaneous biopsy, with minimal trauma to the kidney and the low morbidity associated with laparoscopy. Although laparoscopic surgery is highly efficacious and is associated with high satisfaction rates with minimal morbidity in the management of renal cystic disease[25], a review of the literature revealed only anecdotal case reports of patients with multiple bilateral renal cysts receiving a laparoscopic renal biopsy. Therefore, we believe that patients with polycystic kidney disease who require a kidney biopsy for complex situations will benefit from laparoscopy-guided kidney biopsy with an appropriate risk-benefit ratio. Under this technique, the direct access to the organ permits both selecting the tissue area to be biopsied and attending to any structure injured during the procedure.

In summary, this case underlines the need for renal biopsy in patients with ADPKD and nephrotic-range proteinuria, with or without an accompanying decline in renal function, in order to make an accurate diagnosis and for appropriate treatment/prevention of renal function deterioration. With advances in endoscopic instrumentation, the development of laparoscopic-guided retroperitoneal renal biopsy now provides a minimally invasive alternative to open renal biopsy in the presence of large cysts. To our knowledge, this is the first reported case of nephrotic syndrome due to MN in a patient with ADPKD treated with MFM, with complete remission of nephrotic-range proteinuria and preservation of renal function after more than 10 years.

## ACKNOWLEDGMENTS

This work has been supported in part by grants from the Instituto de Salud Carlos III from Ministerio de Ciencia e Innovación (EC08/00236) and Programa Intensificación Actividad Investigadora (Agencia Laín-Entralgo/CM) to R.P.
